# Research of a 0.14 THz Dual-Cavity Parallel Structure Extended Interaction Oscillator

**DOI:** 10.3390/s24185891

**Published:** 2024-09-11

**Authors:** Chuanhong Xiao, Ruizhe Ren, Zhenhua Wu, Yijun Li, Qing You, Zongjun Shi, Kaichun Zhang, Xiaoxing Chen, Mingzhou Zhan, Diwei Liu, Renbin Zhong, Shenggang Liu

**Affiliations:** 1 School of Electronic Science and Engineering, University of Electronic Science and Technology of China, Chengdu 610054, China; chuanhong_x@163.com (C.X.); 202221020217@std.uestc.edu.cn (R.R.); 202021020527@std.uestc.edu.cn (Q.Y.); shizongjun@uestc.edu.cn (Z.S.); zh.kch@163.com (K.Z.); cxxmarshal@uestc.edu.cn (X.C.); dwliu212220@163.com (D.L.); rbzhong@uestc.edu.cn (R.Z.); liusg@uestc.edu.cn (S.L.); 2 State Key Laboratory of Polymer Materials Engineering, Polymer Research Institute, Sichuan University, Chengdu 610041, China; yijunliruddph@gmail.com; 3 School of Physics, University of Electronic Science and Technology of China, Chengdu 610054, China; mzzhan@uestc.edu.cn

**Keywords:** extended interaction oscillator, terahertz radiation, vacuum electronic device, dual cavity parallel, particle simulation

## Abstract

This paper presents a method to enhance extended interaction oscillator (EIO) output power based on a dual-cavity parallel structure (DCPS). This stucture consists of two conventional ladder-line structures in parallel through a connecting structure, which improves the coupling efficiency between the cavities. The dual output power fusion structure employs an H-T type combiner as the output coupler, which can effectively combine the two input waves in phase to further increase the output power. The dispersion characteristics, coupling impedance, and field distribution of the DCPS are investigated through numerical and simulation calculations, and the optimal operating parameters and output structure are obtained by PIC simulation. At an operating voltage of 12.6 kV, current density of 200 A/${\mathrm{cm}}^{2}$, and longitudinal magnetic field of 0.5 T, the DCPS EIO exhibits an output power exceeding 600 W at a frequency of 140.6 GHz. This represents a nearly three-fold enhancement compared with the 195 W output of the conventional ladder-line EIO structure. These findings demonstrate the significant improvement in output power and interaction efficiency achieved by the DCPS for the EIO device.

## 1. Introduction

The pursuit of high-frequency electromagnetic waves continues to propel technological advancements across various domains. One particularly promising frontier in this endeavor lies within the terahertz (THz) frequency range, spanning from 0.1 to 10 THz. This region of the electromagnetic spectrum holds immense potential for a diverse array of applications, including communication, imaging, sensing, and spectroscopy. However, the effective utilization of the THz regime poses significant challenges, primarily due to the limited availability of a suitable radiation source [1,2,3]. In this paper, vacuum electronic devices (VEDs) emerge as an intriguing avenue, capitalizing on the unique characteristics of VEDs to facilitate high-power, high-frequency operations. Among the notable classes of VEDs, extended interaction oscillators (EIOs) stand out for their exceptional attributes, such as high output power, superior frequency stability, and impressive phase noise performance. Some EIO parameters are shown in Table 1 [4,5,6,7,8].

However, as the frequency increases from the millimeter-wave to the terahertz band, the structure size of the traditional single-cavity EIO decreases, leading to a decline in power capacity that is challenging to improve. In order to solve the limitation of single-cavity EIO power, the combination of multibeam and multicavity is considered. In this case, this paper proposes a dual-cavity parallel structure of EIO, which connects two traditional ladder-line structure EIOs through a rectangular connection structure, and effectively improves the efficiency of the beam-wave interaction. Due to the symmetry of the dual-cavity parallel structure, both cavities can output electromagnetic waves with the same power and frequency, so the power fusion structure is formed through the integration of waveguides, which greatly improves the output power and break through the power limitations of the traditional single-cavity EIOs [9,10,11,12,13,14,15,16,17,18,19,20].

## 2. Structural Design and Cold Cavity Analysis

EIO incorporates a slow-wave structure, exemplified by a ladder-line configuration, as a crucial constituent component, which ensures the coupling impedance, power, and efficiency, while realizing the miniaturization of the device, and also ensures that it is able to interact well with the sheet beam.

The characteristic impedance (R/Q) is a critical factor in measuring the performance of resonant cavities, which can be calculated as follows:

(1) $$\frac{R}{Q}=\frac{{\left(\int_{-\infty }^{\infty }\left|\frac{1}{y}\int_{\frac{-y}{2}}^{\frac{y}{2}}E_{Z}dy\right|dz\right)}^{2}}{2\omega W_{s}}$$

where $E_{z}$ represents the electric field strength in the Z-direction, ω represents the frequency of the resonant cavity, and $W_{s}$ represents the total stored energy.

**Figure 1:**
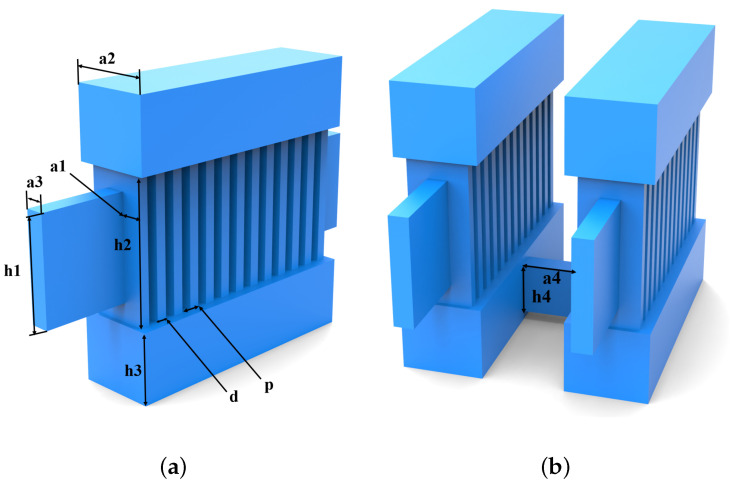
Physical model of the resonant cavity. (**a**) The single-cavity structure. (**b**) The dual-cavity parallel structure.

As illustrated in Figure 1a, the device features a conventional single-cavity structure. Compared to the ${\mathrm{TM}}_{11}$ mode, the ${\mathrm{TM}}_{31}$ mode allows for a more complete interaction between the sheet beam and the electromagnetic field, thereby enabling the achievement of a high output power. To further enhance the output power of the EIO, a dual-cavity parallel structure is proposed in this paper, as shown in Figure 1b, which connects two conventional ladder-line structure EIOs in parallel through a rectangular connecting structure, and this dual-cavity parallel structure EIO not only retains the mechanism of the interaction between the conventional single-cavity structure EIO and the higher order modes as well as the higher efficiency of the beam-wave interactions, but also effectively enhances the coupling efficiency between the cavities. Through extensive theoretical numerical and simulation calculations to optimize the structural parameters, the operating frequency has been determined to be 0.14 THz. The specific parameter values are shown in Table 2.

**Figure 2:**
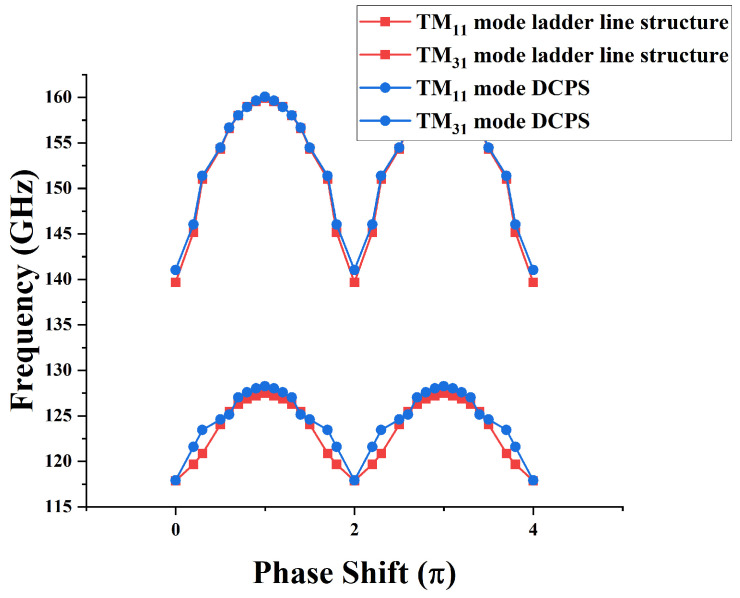
The cold cavity characteristics.

The dispersion curve is obtained as shown in Figure 2 after calculation and analysis. The difference between the dispersion characteristics of the two structures in the same operating mode is not significant. The operating frequency of the ${\mathrm{TM}}_{31}$-2π mode is significantly higher than that of the ${\mathrm{TM}}_{11}$-2π mode, and the operating frequency of the ${\mathrm{TM}}_{31}$-2π mode of the DCPS EIO is 141.03 GHz. In the DCPS EIO, the characteristic impedance of the ${\mathrm{TM}}_{31}$-2π mode (464 Ω) is much larger than that of the ${\mathrm{TM}}_{11}$-2π mode (35 Ω). Therefore, the structure effectively suppresses the ${\mathrm{TM}}_{11}$-2π mode, the ${\mathrm{TM}}_{31}$-2π mode is more easily stimulated for oscillation, and this DCPS has a larger cavity volume and, therefore, a larger power capacity.

**Figure 3:**
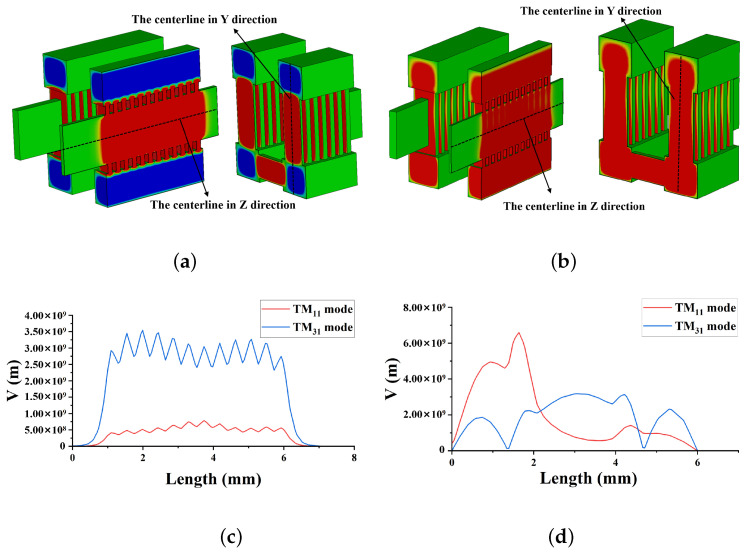
$E_{Z}$ field distribution. (**a**) $E_{Z}$ field distribution in the ${\mathrm{TM}}_{31}$-2π mode. (**b**) $E_{Z}$ field distribution in the ${\mathrm{TM}}_{11}$-2π mode. (**c**) Amplitude of the centerline in the Z-direction. (**d**) Amplitude of the centerline in the Y-direction.

**Figure 4:**
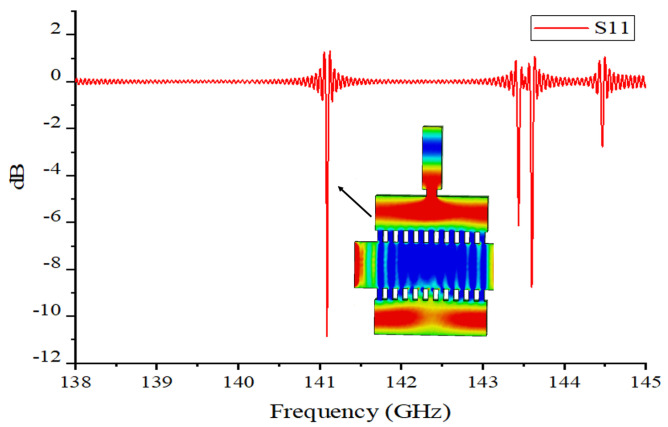
$S_{11}$ parameters of the DCPS EIO and the field distribution of the ${\mathrm{TM}}_{31}$ mode.

The electric field distribution of the two structures of EIO and the $E_{Z}$ electric field strengths measured along the two lines are shown in Figure 3. Figure 3a,b depict the electric field profiles of the ${\mathrm{TM}}_{31}$ and ${\mathrm{TM}}_{11}$ modes, respectively, along with the centerlines in the Y and Z directions. The electric field intensity is then calculated along these central axes. Figure 3c shows the amplitude distribution of the $E_{Z}$ field in the Z-direction. The results show that the $E_{Z}$ field in the gap of the ${\mathrm{TM}}_{31}$ mode is large and stable, and much larger than that of the ${\mathrm{TM}}_{11}$ mode, which indicates that the ${\mathrm{TM}}_{31}$ mode has a stronger beam-wave interaction capability. Figure 3d shows the amplitude distribution of the $E_{Z}$ field in the Y-direction. The results show that the electric field strength in the beam tunnel of the ${\mathrm{TM}}_{31}$ mode is larger than that of the ${\mathrm{TM}}_{11}$ mode, which is favorable to the beam-wave interaction, and that the excessive electric field strength of the ${\mathrm{TM}}_{11}$ mode in the coupling cavity at the lower end may lead to the unbalanced coupling of the electromagnetic wave. Through the cold cavity analysis, the ${\mathrm{TM}}_{31}$ mode was finally selected as the operating mode for the DCPS EIO. The $S_{11}$ parameters of the EIO are shown in Figure 4, from which it can be concluded that there is a good scattering parameter at the output frequency point, the power generated can be well coupled away from the output port.

## 3. PIC Simulation

The optimal structural parameters were determined through cold cavity analysis. To verify the efficacy of the DCPS EIO in enhancing the beam-wave interaction efficiency, PIC simulations were conducted to determine the optimal operating parameters. The size of the electron beam was 2 mm × 0.3 mm, and the current density of each beam was 200 A/${\mathrm{cm}}^{2}$ with an operating voltage of 12.6 kV and longitudinal magnetic field of 0.5 T. Considering the effect of machining roughness and ohmic loss in the DCPS EIO, the Hammerstad-Bekkadal formula can be used to predict the excessive resistance loss and the formula is shown below:

(2) $$\sigma_{eff}=\sigma_{0}\cdot {\left\{1+\frac{2}{\pi }\mathrm{arc}\tan\left[1.4{\left(\frac{h}{\delta }\right)}^{2}\right]\right\}}^{-2}$$

where σ is the conductivity of the ideal smooth surface ($\sigma_{Cu}$ = 5.8 × $10^{7}$ S/m), $\sigma_{eff}$ is the effective conductivity of the rough surface, δ is the skin depth under the ideal smooth plane, and h is the root mean square (RMS) height of the surface. The equivalent conductivity was calculated to be 3 × $10^{7}$ S/m.

**Figure 5:**
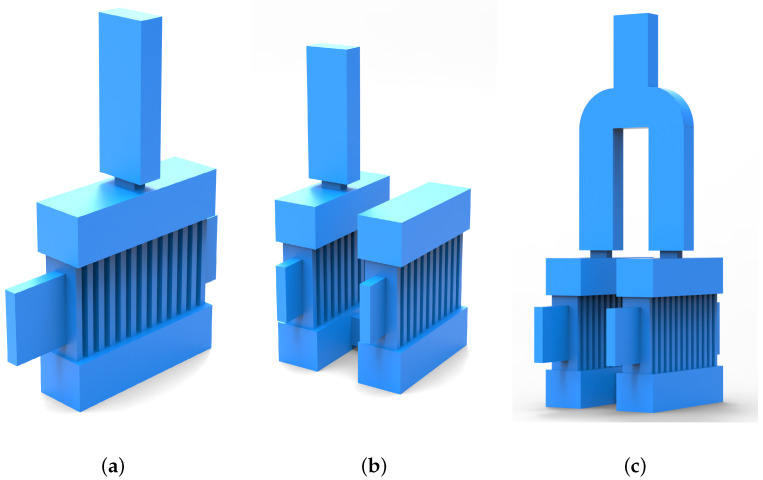
Physical model of the resonant cavity with output structure. (**a**) The single-cavity structure. (**b**) The DCPS structure. (**c**) The DCPS structure with power fusion.

In order to achieve the purpose of significantly increasing the output power, on top of this single output structure, a single output structure with the same structural dimensions was set above both the left and right cavities, and then a 90° curved waveguide was utilized to connect these two single output structures to a rectangular waveguide, which was then connected to the standard waveguide above, to realize the integration of the output structure and to complete the fusion of power. The three structures with output structures are shown in Figure 5.

**Figure 6:**
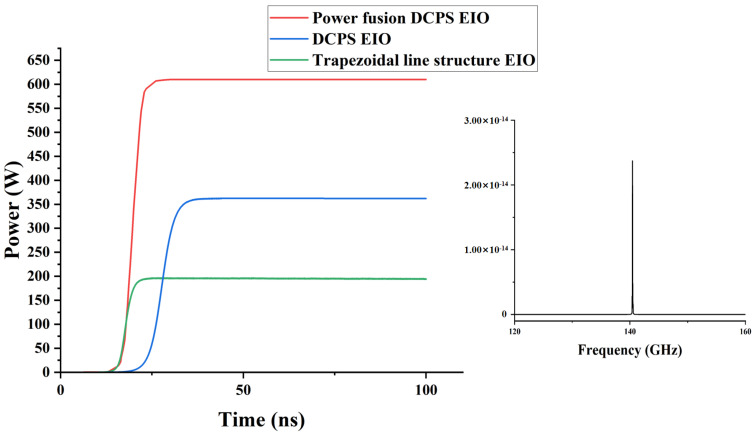
The output power of the three structure’s EIOs and the output frequency of the DCPS EIO.

**Figure 7:**
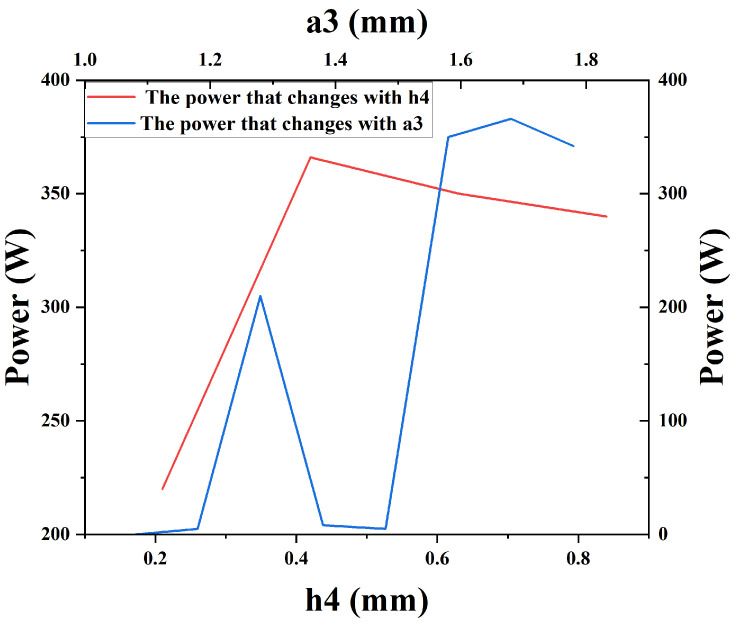
Effect with changing the connection structure on the output power.

**Figure 8:**
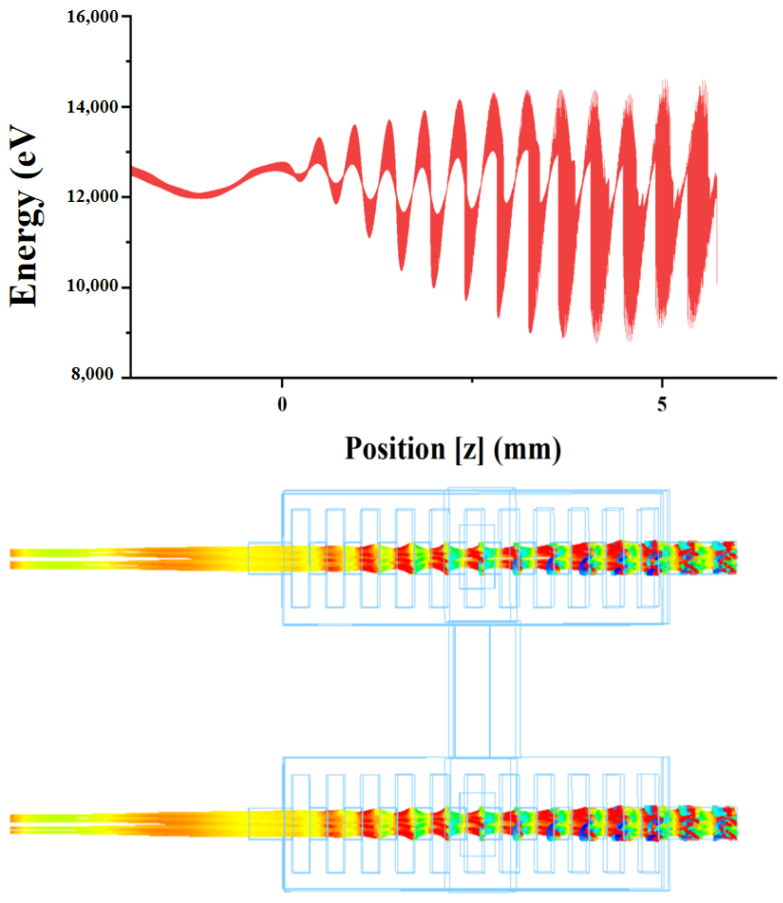
Electron beam energy phase space and cluster.

Figure 6 shows the comparison of the output power of the normal structure, the dual-cavity parallel structure, and the dual-cavity parallel structure with power fusion. Obviously, the output power obtained at 140.6 GHz in the dual-cavity parallel structure was 366 W, which was nearly twice the output power of the normal structure, and both the left and right cavities can output electromagnetic waves, and the output power with power fusion was 610 W. In this study, the coupling of electromagnetic waves was achieved by incorporating a connecting structure between the two cavities. As such, the analysis of the coupling cavity is of critical importance. As shown in Figure 7, the output power results were plotted by changing the length and width of the output cavity. The final dimensions of the coupling cavity were 1.68 mm × 0.44 mm. The phase space of the EIO and the electron beam are shown in Figure 8, where it can be observed that the electron beam was continuously modulated in the resonant cavity and reached the optimal modulation at the end.

## 4. Discussion

To improve the output power of the EIO operating in the terahertz frequency range, this study proposes a new structure called DCPS (dual-cavity parallel structure). DCPS achieves multi-cavity coupling through a coupling structure at the bottom of the cavities. Numerical and simulation analyses of the DCPS demonstrate several key advantages. First, by adopting DCPS, the power capacity is significantly improved compared to the traditional ladder-line EIO structure, providing critical support for the realization of high-power terahertz sources. This is a crucial advancement, as high-power terahertz sources are in high demand for many sensing and imaging applications.

Second, the symmetric design of the DCPS cavities allows for convenient integration of a power fusion structure, such as the H-T type combiner used in this study, to further increase the overall output power. Under the operating conditions of 12.6 kV voltage, 200 A/${\mathrm{cm}}^{2}$ current density, and 0.5 T longitudinal magnetic field, DCPS EIO exhibits an impressive output power exceeding 600 W at a frequency of 140.6 GHz. This represents nearly a three-fold improvement compared with the 195 W output power of the conventional ladder-line EIO. Compared with other EIOs in the same frequency band, the output power is at an optimal level. The significant improvements in output power and interaction efficiency of DCPS can be attributed to the effective coupling between the two parallel cavities and the power combining structure. The design of the electro-optical system will be carried out in a follow-up study and the structure will be processed using the Ultraviolet Lithographie Galvanoformung Abformung (UV-LIGA) technique. This power fusion approach provides a new idea for the subsequent development of high-power terahertz sources.

High-power terahertz sources have a profound impact on the sensor field. For instance, in security screening, the increased output power enables more sensitive and reliable detection of concealed threats, such as explosives and weapons, through enhanced terahertz imaging technology. This capability is particularly valuable in crowded environments, where rapid and accurate screening can significantly enhance public safety. In the medical domain, high-power terahertz sources can improve the quality and penetration depth of imaging, playing a crucial role in the early detection and diagnosis of cancer. By providing more detailed images of tissue structures, these sources can help clinicians identify abnormalities that may be missed with traditional imaging methods. Additionally, these high-power terahertz sources find applications in industrial sectors, such as non-destructive testing and quality control, helping companies ensure the safety and compliance of their products while reducing waste and downtime.

Therefore, I believe that further research on high-power terahertz sources can significantly facilitate the advancement of sensors. This not only enhances the performance of existing technologies, but also drives innovation in new sensor applications, laying a solid foundation for various fields.

## 5. Conclusions

This paper proposes a dual-cavity parallel structure-based extended interaction oscillator that effectively addresses the problem of low output power in traditional single-cavity EIOs at THz frequencies. Under optimal operating conditions, this EIO device achieves an output power exceeding 600 W at 140.6 GHz, nearly tripling the output of conventional EIOs. These findings demonstrate the immense potential of the DCPS approach in developing high-power THz EIO devices and provide an effective technical pathway for the advancement of high-power THz sources.

## Figures and Tables

**Table 1:** Parameters of the current EIOs.

Institution	Frequency	Voltage	Current	Power
	(GHz)	(kV)	(A)	(W)
CPI	93.8	20.3	0.69	1400
UESTC	140	18	1.5	661
CPI	140	/	/	200
UESTC	220	16.6	3.2	500
CPI	214.5	11	0.095	13.3
UESTC	300	14.8	0.25	250

**Table 2:** Parameters of the resonant cavity.

Parameter	Quantity	Value (mm)
a1	Gap depth	0.42
a2	Coupling cavity width	1.68
a3	Beam tunnel width	0.40
a4	Coupling cavity width	1.68
h1	Beam tunnel height	2.00
h2	Gap height	3.00
h3	Coupling cavity height	1.50
h4	Coupling cavity height	1.10
d	Gap width	0.22
p	Period length	0.44

## Data Availability

The data that support the findings of this study are available from the corresponding author upon reasonable request.
